# Unicentric Castleman disease in intraparotid lymph node: a case report

**DOI:** 10.1186/s40902-025-00491-8

**Published:** 2025-11-11

**Authors:** Young Heon Jeong, Jin Seok Kim, Heonwoo Lee, Kang-Min Ahn

**Affiliations:** 1 Department of Oral and Maxillofacial Surgery, College of Medicine, University of Ulsan, Seoul, Republic of Korea; 2 Department of Pathology, College of Medicine, University of Ulsan, Seoul, Republic of Korea

**Keywords:** Castleman disease, Lymphadenopathy, Parotid gland, Lymph nodes, Facial nerve

## Abstract

**Background:**

Castleman disease (CD) is a rare lymphoproliferative disorder characterized by non-neoplastic lymph node hyperplasia. Unicentric Castleman disease (UCD), the most common clinical form, typically presents as a solitary, asymptomatic mass. Involvement of intraparotid lymph nodes is rare and often mimics salivary gland neoplasms, complicating preoperative diagnosis.

**Case presentation:**

A 35-year-old female presented with a painless, enlarging mass in the left parotid region. Contrast-enhanced CT revealed a well-demarcated, homogeneously enhancing mass with additional smaller lesions in adjacent lymph node regions. A conservative excisional approach was performed with preservation of the facial nerve. Histopathological evaluation confirmed the hyaline-vascular variant of UCD. Postoperative follow-up showed spontaneous regression of adjacent lymphadenopathy and resolution of transient facial nerve palsy.

**Conclusion:**

CD should be considered in the differential diagnosis of encapsulated parotid masses with associated lymphadenopathy. Recognizing its clinical and radiologic features may facilitate accurate diagnosis and prevent overtreatment. Complete surgical excision is curative in most UCD cases.

## Background

Castleman disease (CD), first described by Dr. Benjamin Castleman in 1956, represents a rare group of lymphoproliferative disorders characterized by non-neoplastic lymph node hyperplasia with inflammatory symptoms and laboratory abnormalities [1]. It shows polyclonal B lymphocyte proliferation with uncertain malignant potential [2]. CD is clinically classified into two primary categories based on the extent of lymph node involvement: unicentric Castleman disease (UCD) and multicentric Castleman disease (MCD) [3]. UCD in the head and neck typically presents as an asymptomatic, slowly growing neck mass, often discovered incidentally [4]. UCD usually involves a single lymph node, or occasionally, one group of lymph nodes [5]. In contrast, MCD may present with multiple bilateral enlarged lymph nodes accompanied by systemic symptoms including thrombocytopenia, anasarca, fever, renal dysfunction, organomegaly [4, 6], MCD is classified into two subtypes based on Human Herpes virus-8 (HHV-8) association: MCD, idiopathic(iMCD) and MCD, HHV8-associated (HHV8-MCD) [7]. According to various studies, UCD accounts for up to 80% of all CD cases, while MCD comprises approximately 20–25% [4]. Histologically, CD can be classified into two variants: the hyaline vascular (HV) type and the plasma cell (PC) type [8, 9]. There is a strong association between these histologic subtypes and their clinical manifestations [9]. Reports indicate that 65% to 80% of UCD cases exhibit features of the HV type, whereas the remaining 20% to 35% correspond to the PC type. In contrast, the majority of MCD cases (over 90%) display histologic characteristics of the PC variant [8, 10]. In the treatment of CD, complete surgical excision is often sufficient for UCD except symptomatic PC-CD which could require systemic treatment. On the other hand, MCD often has an unfavorable prognosis, especially in HIV-positive patients and those with POEMS syndrome [6]. Therefore, treatment for MCD typically involves systemic therapy, including corticosteroids, various immunosuppressive or immunomodulatory agents, cytotoxic chemotherapy, rituximab (an anti-CD20 antibody), siltuximab (an anti–IL-6 antibody), and tocilizumab (an anti–IL-6 receptor antibody). [4]

In this case report, we present a case of CD arising in a lymph node in the parotid gland. Preoperative evaluation with computed tomography (CT) showed encapsulation of the mass, raising the possibility of a salivary gland origin tumor. Although lymph node enlargement raised the possibility of malignancy, surgical excision was performed conservatively to preserve function and minimize complications, with the possibility of further surgery if malignancy was later established. Histopathological examination confirmed CD, indicating that appropriate surgical management had been performed.

## Case presentation

**Fig. 1:**
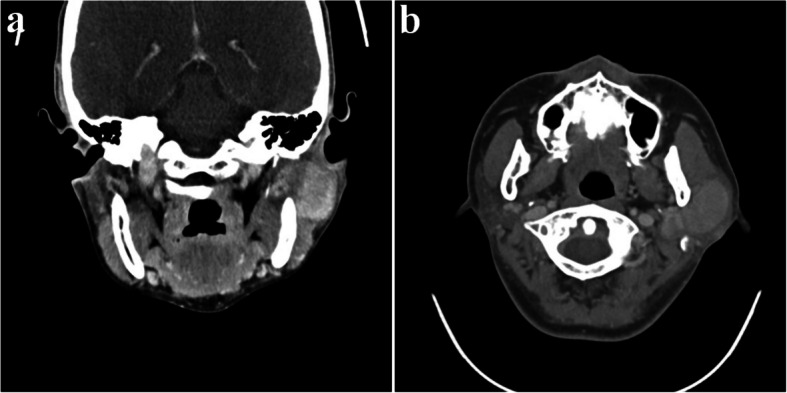
Preoperative CT images (parotid gland area); a well-defined mass in the left parotid gland with homogeneous strong enhancement. **a** Coronal view, neck extension, **b** axial view

**Fig. 2:**
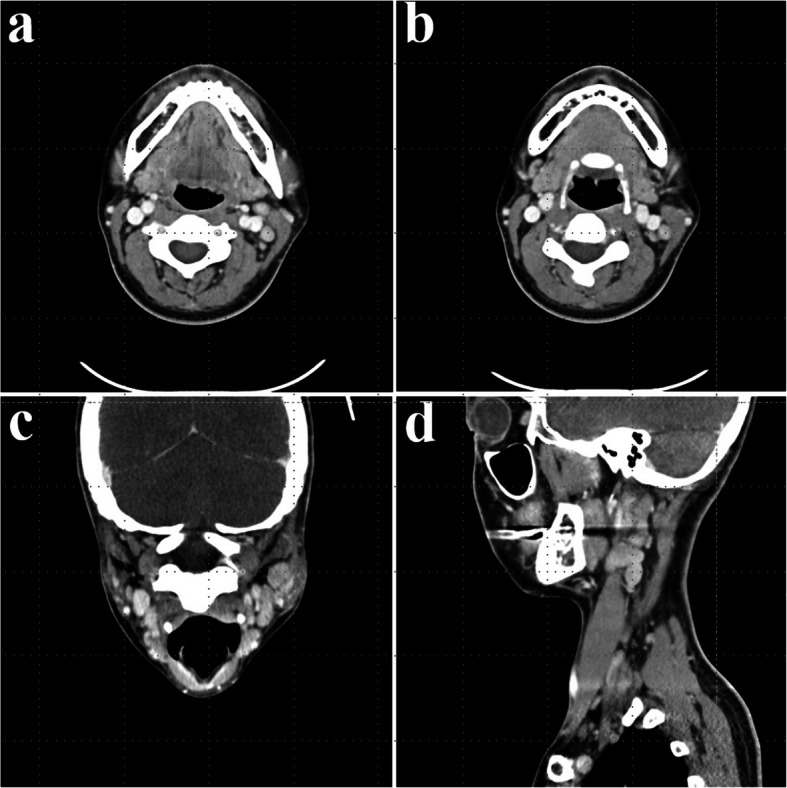
Preoperative CT images (lymph node area); multiple smaller lesions exhibiting similar enhancement patterns in the left level II, III). **a**, **b** Axial view, **c** coronal view, neck extension, **d** sagittal view

A 35-year-old female presented with swelling on the left side of her face for approximately 3 months. Contrast-enhanced CT showed a well-circumscribed, homogeneously enhancing mass in the left parotid gland, with additional smaller lesions of similar characteristics identified in the left parotid deep lobe, as well as in the left level II and right level VI regions (Fig. 1). Multiple small reactive lymph nodes were also noted in the left level III, IV, and V regions (Fig. 2). Although such findings can be suggestive of malignancy, we decided to remove the mass in a conservative approach considering the patient’s age and the potential functional impairments associated with radical surgery, like facial palsy. Informed consent was obtained, including agreement for further resection if histopathological examination revealed malignancy.

**Fig. 3:**
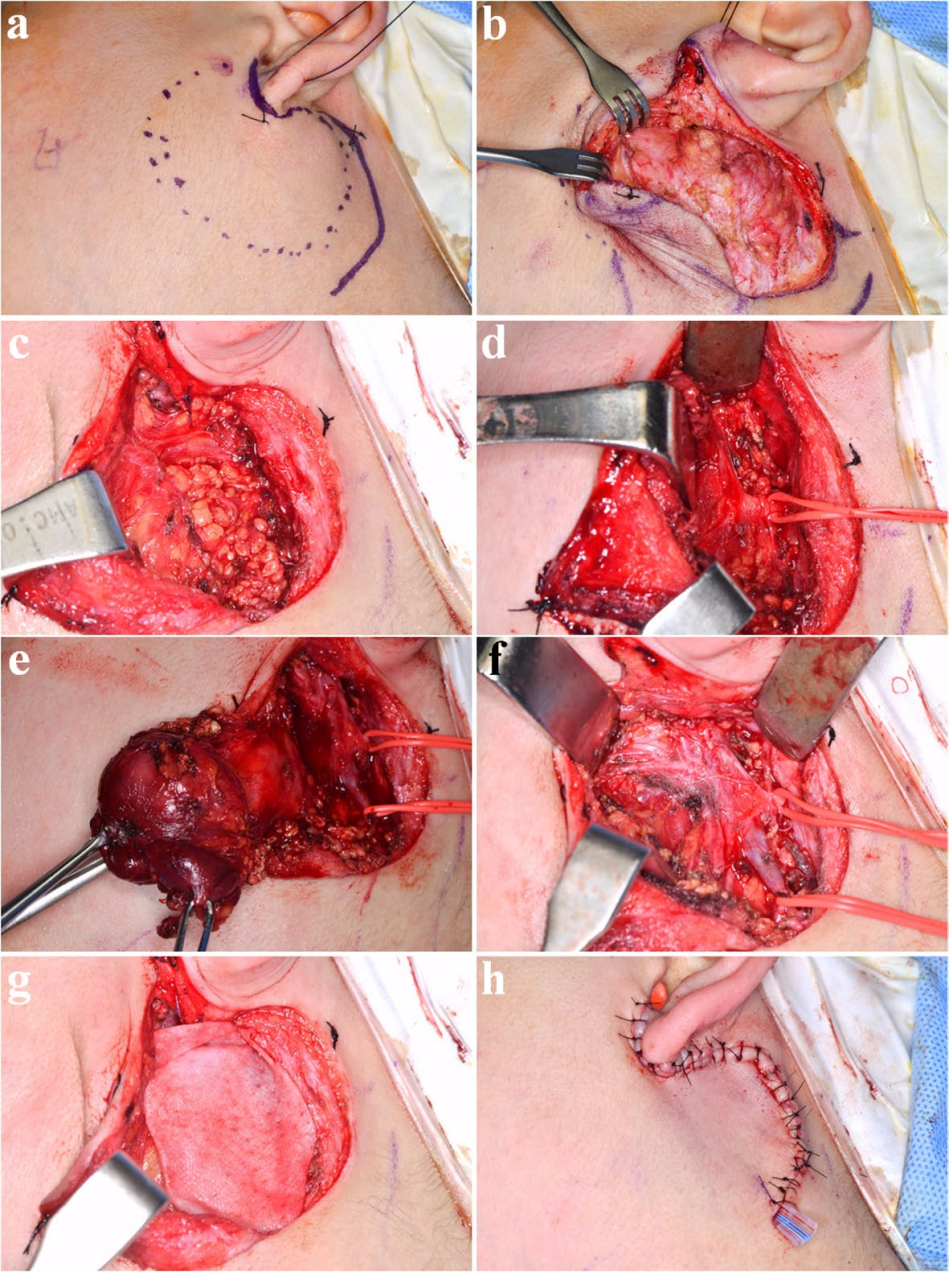
Total parotidectomy. **a** Preoperative clinical photograph. **b** The retromandibular incision with extension to the retro-auricular area, anterior to the ear lobe, was made, and the SMAS layer was exposed. **c** Exposure of the parotid gland. **d** Identification and marking of the facial nerve trunk. **e** Retraction of the superficial lobe of the parotid gland containing the mass. **f** After total parotidectomy, with preservation of the facial nerve and retromandibular vein. **g** Application of ADM. **h** Wound closure completed with drain insertion

**Fig. 4:**
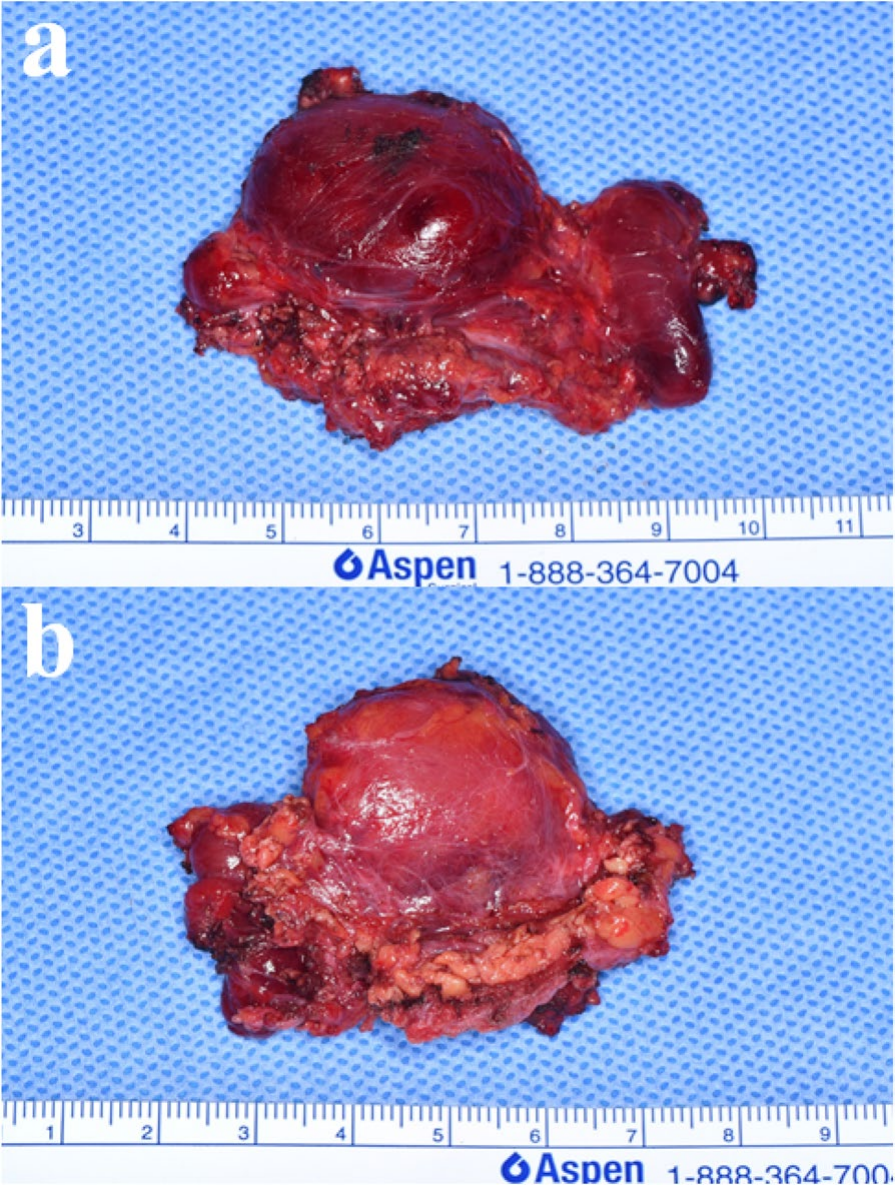
Clinical photograph of the surgical specimen following total parotidectomy. **a** Inner surface. **b** Outer surface

The surgery was done under general anesthesia. The retromandibular incision with extension to the retro-auricular area, anterior to the ear lobe, was done (Fig. 3a). Careful dissection was done. After the subcutaneous tissue was undermined, the superficial musculo-aponeurotic system (SMAS) was exposed (Fig. 3b). A vertical incision was made through the SMAS into the parotid gland (Fig. 3c). The first aim of this stage was to expose and preserve the facial nerve trunk without impairment (Fig. 3d). The parotid gland was bluntly dissected parallel to the direction of the facial nerve branches and towards the anterior border of the parotid gland. The dissection was done from the retromandibular vein to the anterior (Fig. 3e). Branches of the facial nerve were found during the dissection (Fig. 3f). The facial nerve trunk was identified and protected. After preserving the facial nerve, the total parotid gland, including the mass, was dissected and excised (Fig. 4). Saline irrigation and hemostasis were done. The cadaveric acellular dermis matrix (Megaderm®) was inserted for prevention of fibrosis and scar tissue formation (Fig. 3g). Suture was done with drain insertion (Fig. 3h).

**Fig. 5:**
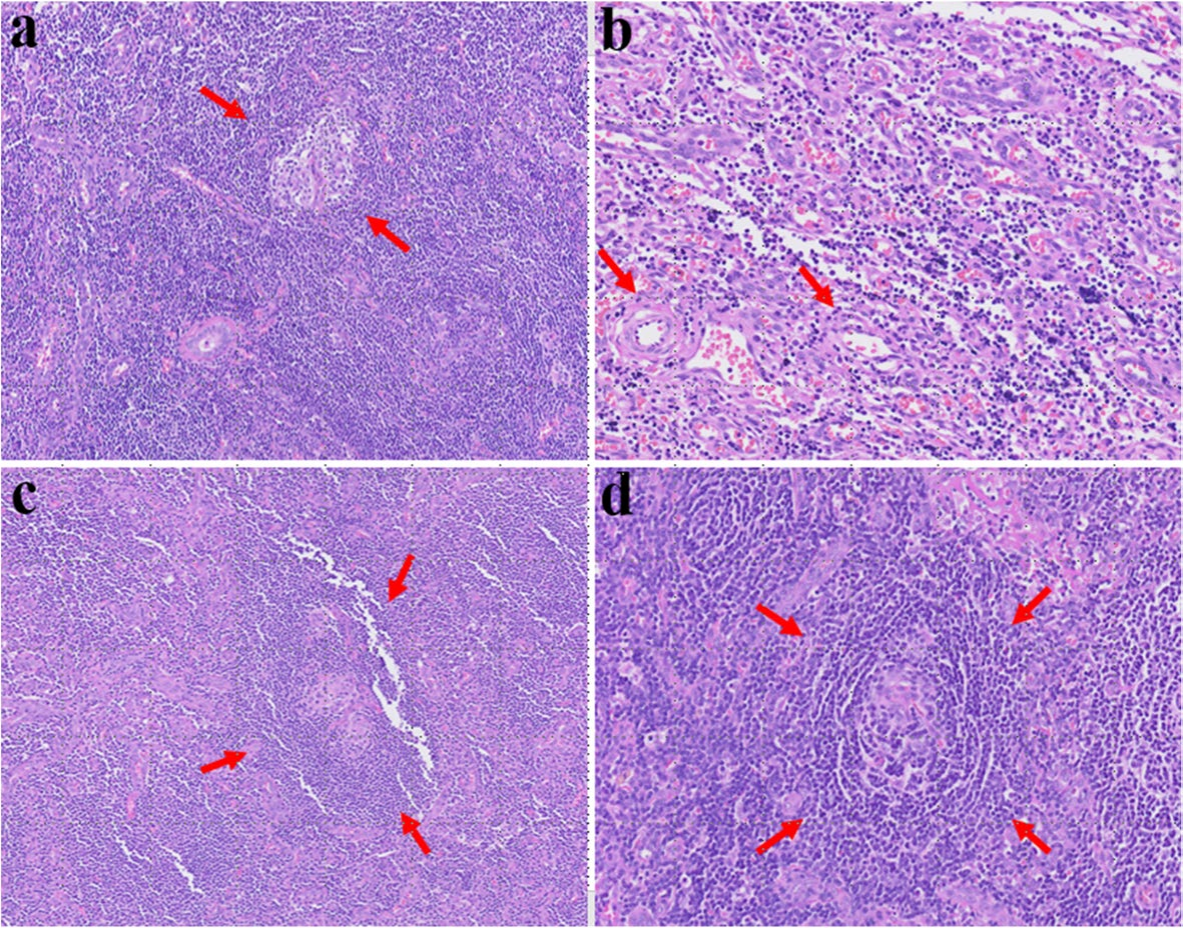
Microscopic images showing HV-CD features. **a** Atrophic germinal center and a radially traversing hyalinized blood vessel (Lollipop sign). **b** proliferation of high endothelial venules (HEV). **c **Two small germinal centers (twining appearance). **d** Expanded mantle zone with concentric arrangement (onion skin appearance)

All of frozen biopsy during surgery was negative for malignancy. Final histopathological diagnosis was CD, hyaline-vascular variant. It showed an enlarged intra-parotid lymph node with severe paracortical hyperplasia and atrophic germinal centers (Fig. 5). Immunohistochemistry for HHV-8 was performed, which did not demonstrate HHV-8 expression. After diagnosis, the patient was referred to the oncology department for evaluation of the need for chemotherapy. CT scans of the neck, chest, abdomen, and pelvis were performed, along with whole-body PET/CT, which confirmed the diagnosis of UCD. The oncologist decided to proceed with regular follow-up without chemotherapy. Stitch-out was performed after 8 days. At 4 months after surgery, the scar was minimal and favorably hidden within the hairline (Fig. 9a). As preservation of the facial nerve was confirmed during surgery, the patient was managed with observation alone, without specific treatment. The facial weakness gradually subsided, and the palsy had completely recovered by 4 months after surgery. Preservation of the temporal and zygomatic branches of the facial nerve was confirmed through forceful eye closure, and the functions of the buccal and marginal mandibular branches were verified during smiling (Fig. 9b, c). At the current follow-up, 4 months after surgery, a low-dose CT scan was performed, which revealed no evidence of recurrence or pathologic lymph nodes. The patient will undergo annual low-dose computed tomography (CT) scans of the neck, chest, abdomen, and pelvis for periodic surveillance.

## Conclusion

UCD rarely arises within intra-parotid lymph nodes, making its clinical recognition particularly challenging, as such lesions often mimic salivary gland neoplasms on imaging [11]. By definition, UCD encompasses both the enlargement of a solitary lymph node and the involvement of multiple lymph nodes within a single region [5]. In this case, the main parotid mass was accompanied by multiple regional lymphadenopathies in adjacent areas, a finding that initially raised concern for malignancy. Following total parotidectomy, the accompanying regional lymphadenopathies underwent spontaneous regression, a rare observation in previously reported cases that emphasizes the distinctive clinical characteristics of this presentation [12, 13].

In the head and neck area, CD typically presents as a solitary, oval-shaped, firm, and non-tender mass without signs of inflammation. Radiologic evaluation contributes to differential diagnosis. On contrast CT, the lesion appeared as a densely enhancing, oval-shaped, homogeneous mass, suggesting pronounced vascularity [14]. The hyaline-vascular type shows more pronounced enhancement due to its hypervascularity [15]. Although magnetic resonance imaging (MRI) features are not specific, CD should be included in the diagnostic consideration in cases of a discrete cervical mass exhibiting high signal intensity on T2-weighted images and low to intermediate signal intensity on T1-weighted images [16]. In the present case, preoperative evaluation was done using only contrast-enhanced CT. The presence of multiple enlarged lymph nodes initially raised suspicion for malignancy; however, the findings of homogeneous and dense enhancement, particularly in multiple nodes located at the same level, suggest the possibility of UCD. Therefore, UCD should be considered in the differential diagnosis under such radiologic circumstances.

**Fig. 6:**
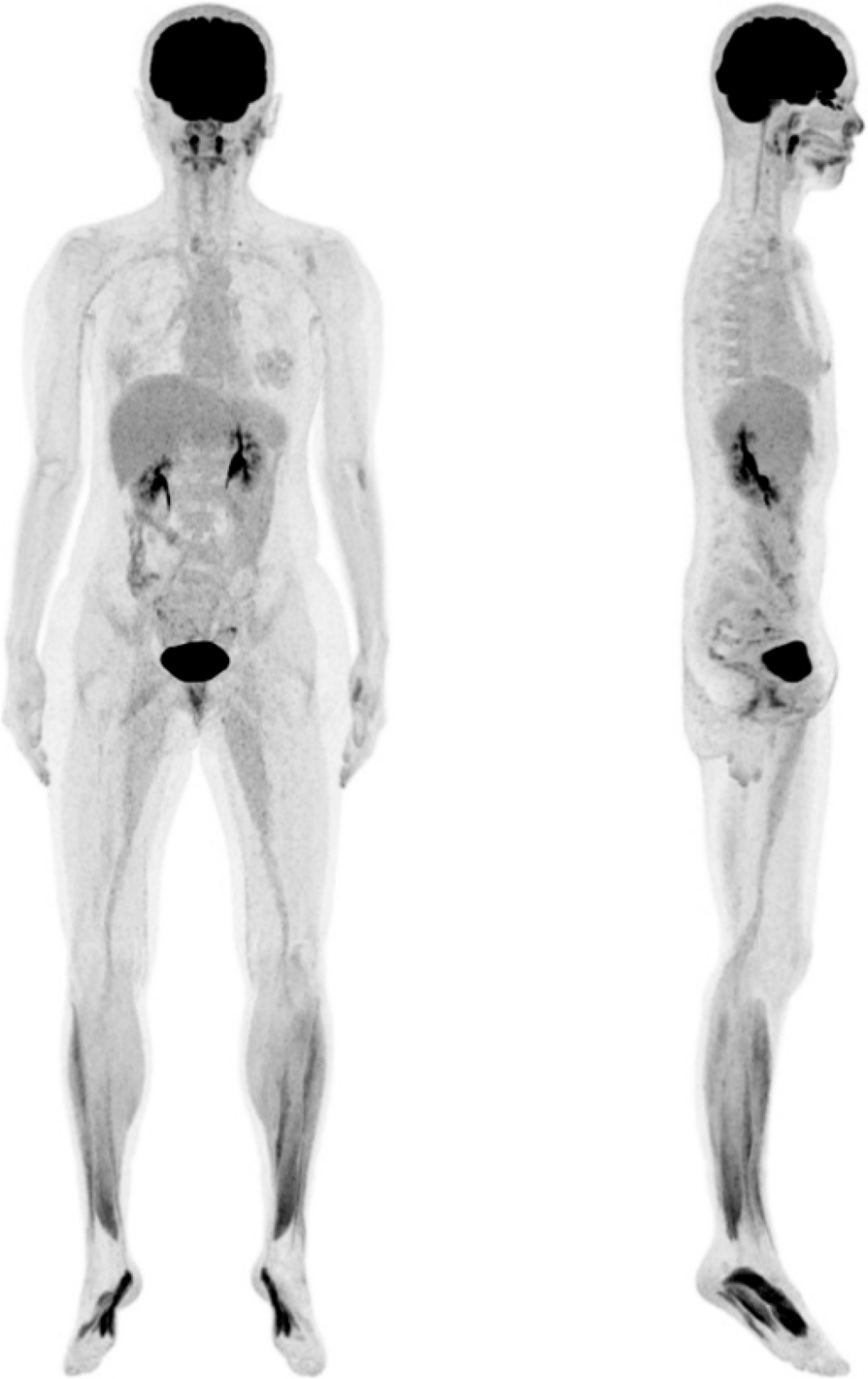
Whole body PET/CT images

In our case, the hyaline-vascular (HV) subtype of Castleman disease presented as a localized, asymptomatic lesion. This variant can occur across a wide age range and shows no significant gender predominance. Histologically, HV-CD is characterized by preserved but distorted lymph node architecture with several features. The germinal centers are typically atrophic and penetrated by radially oriented sclerotic blood vessels, forming the classic “lollipop sign” (Fig. 5a). There is often prominent proliferation of high endothelial venules (HEV) throughout the interfollicular regions which reflects abnormal angiogenesis and immune cell trafficking (Fig. 5b). Another distinctive finding is follicular twinning, in which a single follicle contains two small germinal centers (Fig. 5c). Surrounding mantle zones are notably expanded and composed of concentrically arranged small lymphocytes, creating the “onion-skin” appearance (Fig. 5d). These combined features are pathognomonic of the hyaline-vascular variant and support the diagnosis (Fig. 6).

**Fig. 7:**
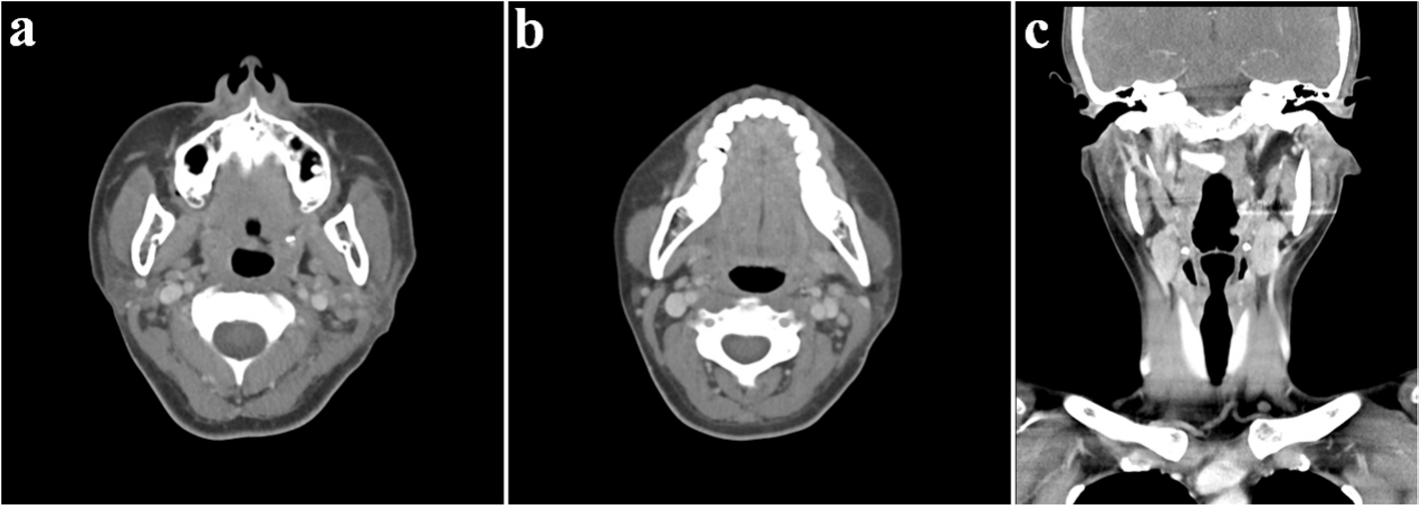
Postoperative CT images on day 8. **a** Axial view (parotid gland area). **b **Axial view (lymph node area, level II, III); decreased size of the lymph nodes. **c **Coronal view

**Fig. 8:**
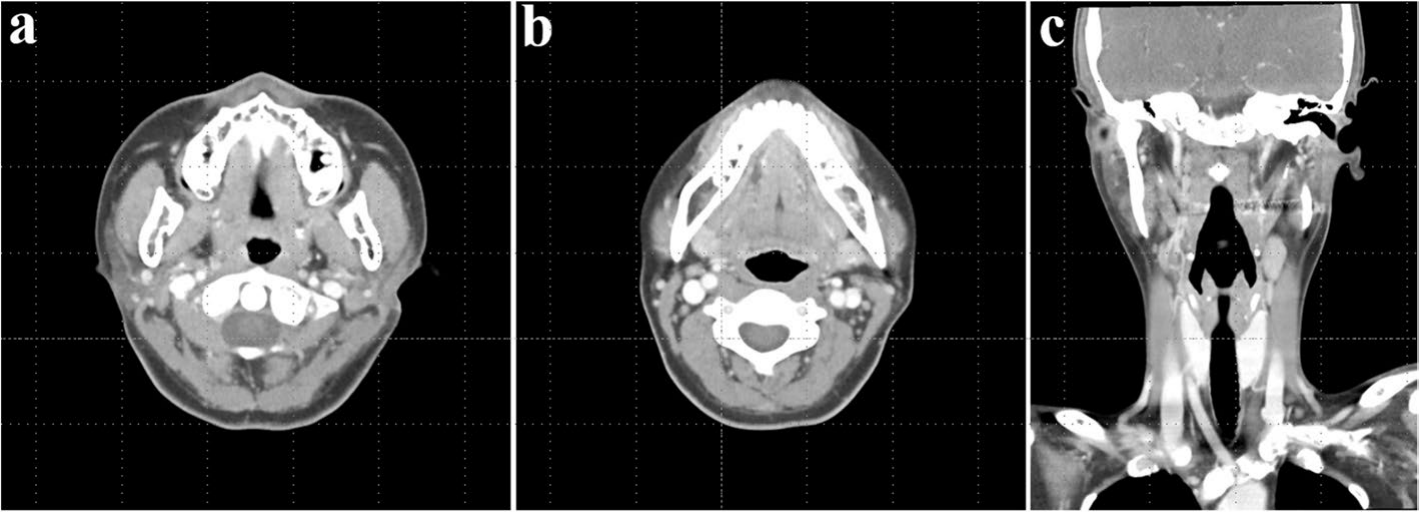
Postoperative CT images at 4 months. **a** Axial view (parotid gland area). **b** Axial view (lymph node area, level II, III); no pathologic lymph nodes. **c **Coronal view

The prognosis of CD varies significantly depending on the clinical type and treatment approach [4, 17]. In UCD, surgical excision is the primary treatment, regardless of histologic subtype [17]. In the systematic review of 404 published cases of CD, surgical resection was shown to be highly effective for UCD (UCD), with a reported overall survival rate of 95.3% [5]. If surgery is impossible, treatment alternatives may include irradiation, embolization, or neoadjuvant therapy using rituximab or siltuximab/tocilizumab in the presence of acute inflammation [4]. Radiation therapy can also be effective as a primary treatment, but it is usually limited to select patients who are not candidates for surgery due to potential risks [17]. In cases of UCD presenting with enlargement of multiple lymph nodes within a single region, small regional satellite lymph nodes typically regress following surgical removal of the primary lesion [18]. In this case as well, postoperative CT obtained on day eight demonstrated a decrease in the size of lymph nodes in the left level II–V and right paratracheal areas, supporting spontaneous regression following excision of the primary lesion (Figs. 7 and 8).

**Fig. 9:**
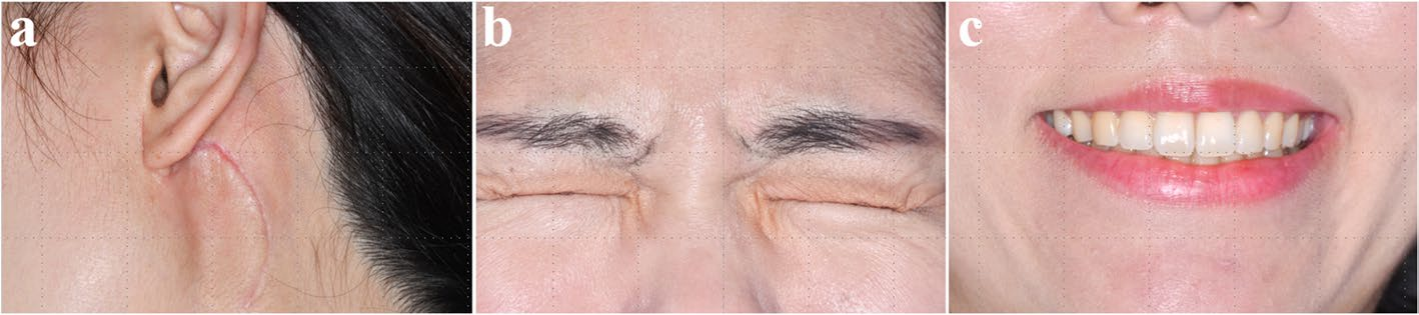
Postoperative clinical photographs at 4 months. **a** Surgical site. **b** Preserved temporal branch and zygomatic branch function. Preserved marginal mandibular and buccal branch functions

There was only a temporary symptom of the facial nerve, which recovered without any permanent damage. Although malignancy was suspected preoperatively, the surgery was performed with facial nerve preservation in mind, considering the possibility of reoperation. This approach led to a favorable outcome (Fig. 9b, c). To ensure nerve preservation, an anterograde dissection technique was employed. The facial nerve trunk was first identified at the posterior border of the parotid gland, followed by careful identification and dissection of each peripheral branch. Gentle tissue handling and meticulous hemostasis were maintained throughout the procedure. Fine, atraumatic instruments were used, and excessive retraction or electrocautery near the nerve branches was avoided to minimize the risk of iatrogenic injury.

In this surgery, we used cadaveric skin-derived acellular dermal matrix (ADM) to prevent and reduce specific post-surgical complications. There are three reasons for using ADM. The first reason is that ADM acts as a physical barrier that prevents aberrant regeneration of parasympathetic nerve fibers, which can otherwise connect with sweat glands and cause gustatory sweating, known as Frey’s syndrome [19, 20]. The second reason is the improved cosmetic outcome. By occupying the surgical defect, ADM helps prevent infra-auricular (near the ear) depressed deformities and facial contour irregularities, leading to higher patient satisfaction with their facial appearance [21]. The third reason is the reduction in First Bite Syndrome and Acute Pain. ADM can reduce the incidence and severity of first bite syndrome (sharp pain during the initial mastication of food) and post-surgical discomfort. Also, in this case, it could be helpful for further resection if the final diagnosis was malignant.

Unlike the submandibular gland and the sublingual gland, the parotid gland has lymphoid tissue within the glandular tissue. In 2014, Ergün et al. investigated 84 parotid glands and reported that intra-parotid lymph nodes were identified in the superficial lobe in 95% of the cases and in the deep lobe in 31%. On average, 2.97 lymph nodes were found in the superficial lobe and 0.41 in the deep lobe. Overall, 88% of cases exhibited lymph nodes within the superficial lobe [22]. Considering that lymph nodes may be present within the parotid gland, especially in the superficial lobe, it is important to consider that masses identified on imaging may arise from intra-parotid lymphoid tissue rather than from salivary epithelial origin. This distinction is particularly relevant in rare cases such as CD. When evaluating an encapsulated or lymph node-like lesion in the parotid region with associated lymphadenopathy, clinicians should include CD in the differential diagnosis, especially the hyaline-vascular variant, which tends to present as a solitary, well-defined mass. Awareness of this possibility may prevent unnecessary radical surgery and guide appropriate diagnostic and therapeutic planning.

## Data Availability

No datasets were generated or analysed during the current study.

## Abbreviations

CD: Castleman disease
UCD: Unicentric Castleman disease
MCD: Multicentric Castleman disease
HV: Hyaline vascular
PC: Plasma cell
iMCD: Idiopathic multicentric Castleman disease
HHV-8: Human herpes virus 8
CT: Computed tomography
MRI: Magnetic resonance imaging
SMAS: Superficial musculo-aponeurotic system
ADM: Acellular dermal matrix
